# Supplementary material for: Beyond the healthcare system: The societal and contextual factors impacting parents’ participation in decision-making for neonates with life-threatening conditions
Source: PLoS One. 2024 Sep 6;19(9):e0309914. doi: 10.1371/journal.pone.0309914 (PMC11379267; doi:10.1371/journal.pone.0309914)
Supplement: Attachment — Submitted filename: corrections PLOS ONE.docx [file pone.0309914.s001.docx]

**Reply to comments** ID PONE-D-24-09858

Translation and psychometric evaluation of the Persian version of the Nurse Clinical Reasoning Scale: A methodological study

First of all, we would like to express our gratitude to the editor and the reviewers for their constructive comments and helpful suggestions. We greatly appreciate the careful reading of our manuscript. We have considered your comments and corrected the manuscript accordingly. A point-by-point response to the referees’ comments is given below:

If you have any questions about this paper, please do not hesitate to let me know. My e-mail address is:

banazadehmarjan@gmail.com

| Comments | Ref/ Page |
| --- | --- |
| Reviewer #1: A brief description of the local health system structure is necessary since in the results it appears as one of the analysis categories and the American System is mentioned in the discussion to compare. | I would like to express my gratitude to the esteemed reviewer for their insightful comments.  The recommended information was added and the highlighted details can be found on Pages 17, lines 13-23, 18, lines 13-23,19, and lines 1-6. |
| Reviewer #2: The study provides a good contribution regarding factors that impact parental participation in medical decisions related to critically ill newborns and this aspect is best addressed with qualitative research. Despite the small sample, all steps of the method are adequately described, with a correct explanation of possible biases. The results adequately support the conclusions and achieve the proposed objectives. | I would like to express my gratitude to the esteemed reviewer for their insightful comments. |
